# Tissue engineering of the retina: from organoids to microfluidic chips

**DOI:** 10.1177/20417314211059876

**Published:** 2021-12-10

**Authors:** Luis F Marcos, Samantha L Wilson, Paul Roach

**Affiliations:** 1 Department of Chemistry, School of Science, Loughborough University, Leicestershire, UK; 2 Centre for Biological Engineering, School of Mechanical, Electrical and Manufacturing Engineering, Loughborough University, Leicestershire, UK

**Keywords:** Biofabrication, biomaterials, microfluidics, retina, retina-on-a-chip, retinal organoids, retinal tissue engineering

## Abstract

Despite advancements in tissue engineering, challenges remain for fabricating functional tissues that incorporate essential features including vasculature and complex cellular organisation. Monitoring of engineered tissues also raises difficulties, particularly when cell population maturity is inherent to function. Microfluidic, *or lab-on-a-chip*, platforms address the complexity issues of conventional 3D models regarding cell numbers and functional connectivity. Regulation of biochemical/biomechanical conditions can create dynamic structures, providing microenvironments that permit tissue formation while quantifying biological processes at a single cell level. Retinal organoids provide relevant cell numbers to mimic in vivo spatiotemporal development, where conventional culture approaches fail. Modern bio-fabrication techniques allow for retinal organoids to be combined with microfluidic devices to create anato-physiologically accurate structures or ‘*retina-on-a-chip’* devices that could revolution ocular sciences. Here we present a focussed review of retinal tissue engineering, examining the challenges and how some of these have been overcome using organoids, microfluidics, and bioprinting technologies.

## Introduction

Retinal degeneration encompasses a range of different pathologies at the molecular, cellular and tissue level including environmental, inherited, or age-related diseases, for example, retinitis pigmentosa (RP) and age-related macular degeneration (AMD). Such diseases involve the damage and eventual loss of retinal cells, specifically photoreceptors and the retinal pigmented epithelium (RPE) and are the leading cause of vision impairment and blindness worldwide; globally, AMD ranks third after cataracts and glaucoma and is the primary cause of blindness in industrialised countries.^1^ RP is one of the most prevalent inherited retinopathies, affecting 1 in 3500 to 1 in 4000 people worldwide.^2^ Regardless of the initial cause of retinal degeneration, if the photoreceptor-RPE system is disturbed, photoreceptors lose their outer segments and cellular stress pathways are engaged. This leads to cell death and loss of photoreceptors resulting in an inevitable progressive sequence of negative neuronal remodelling and restructuration of the retina, resulting in vision loss and irreversible blindness.^3^

Due to the inherent lack of regenerative capacity of the mammalian retina, diverse therapeutic concepts are under investigation. One of the first strategies to be developed and to receive regulatory approval consisted of electric retinal prostheses to replace lost photoreceptor function.^4^ Although such technology-enhancements are promising, in addition to current gaps in the understanding of retinal processing, they are limited in their visual resolution capabilities and difficulties concerning integration with the biological system.^4,5^ Advances in molecular genetics and cell biology are clarifying the pathophysiological mechanisms driving retinal disorders and identifying new approaches such as cell, gene and optogenetic therapies.^6^ Whilst all these approaches present their own advantages and disadvantages, different prosthetic devices such as the Alpha IMS and Argus II^7,8^ are currently commercially available, and therapeutic strategies are progressing through the clinical phases of development.^1^

Dependent upon the severity of a disease and its developmental stage, the use of one therapeutic strategy may be more appropriate than others. Critical to retinal pathologies, some strategies aim to recover the remaining retinal cells, whilst others aim to replace the lost cell types.^9^ Cell transplantation approaches for the replacement of lost photoreceptors is a major goal in the research community and has been investigated for the last three decades.^10^ Despite huge developments in the field, finding the optimal cell delivery method, successful integration into the host retina, and reconstruction of the neural circuitry and functionality are the major hurdles for successful cell transplantation.^10^ Nevertheless, no definitive cure for retinal pathologies has been established and conventional therapies/research models in use have important limitations, presenting barriers to effective translation.

Tissue engineering approaches have rapidly evolved over the last 20 years, enabled by emerging technologies and cellular understanding. In vitro fabrication of organised cell networks has been used to either present a model tissue, for example, for drug testing and have been a focus for tissue engineers. Often these models make use of 2D culture systems, which are often limited in the number of cells and cell types used which ultimately impacts tissue maturation, resulting in the complexity of the tissue formed being distinctly different to native tissue. Advances in 3D culture technologies and tissue engineering techniques have overcome some of these obstacles, although at present, challenges remain in terms of fabrication variability, consistency and robustness. There are also limitations in the methodologies used to interrogate cell culture systems, increasing with tissue complexity. Currently, developments in *organ-on-a-chip* systems have increased tissue-specific in vitro models for a range of tissues/organs (e.g. *heart-on-a-chip* or *lung-on-a-chip*^11^) mimicking their primary functions. However, such technology is still far from mirroring more complex system, such as neurological tissues, that is, the retina. Further advances in biofabrication and *on-chip* devices research would likely consolidate existing models and generate complex neural tissue structures bearing higher fidelity. Ultimately, such tools would be useful for probing disease-specific mechanisms, facilitating development of novel therapeutics and promoting neural regeneration.^12^ Here we review some of the more recent advances in retinal tissue engineering, as well as the challenges faced, in terms of functional complexity, the current situation and future perspectives including microfluidics, retinal organoids and bioprinting technologies.

### Tissue engineering of the neural niche

**Figure 1.**
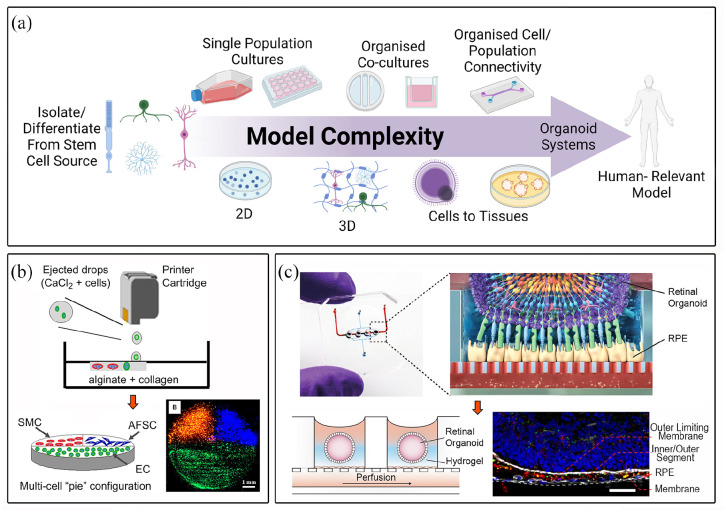
Representation of increasing complexity parameters to define appropriate cell/tissue models (a), multicellular bioprinted platform (b; 13), combination of retinal organoids and microfluidics technologies (c; 39). Technology advances have allowed the rapid progression from 2D to more complex and representative 3D in vitro models. Fine control over the materials and the precise allocation of different cell types through a variety of biofabrication technologies, results in the establishment of functional interconnectivity between the cells, leading towards the generation of functional in vitro living 3D structures. Furthermore, novel combination of tissue engineering and biofabrication strategies, can overcome some of the most common drawbacks that 3D models normally present. Reproduction of images are under copyright: (b) Elsevier, Xu et al.^13^ (c) Creative Commons Attribution 4.0 International License (https://creativecommons.org/licenses/by/4.0/legalcode), Achberger et al.^39^ created with BioRender.com.

There are an array of 3D tissue engineering and biofabrication techniques allowing control over the final material with precise location of many different cell types and surrounding extracellular matrix (ECM) mimicking biomaterials. These have developed with a focus on the technological capability, for example, multiple simultaneous bioprinting of many cell types into a single tissue, to generate artificial living 3D structures.^13^ The complexity of the formed tissue and its function is underpinned by the cellular presentation within its matrix, which enables any resulting interconnectivity between cells. Complexity of tissue is therefore defined by the different types of cells held, within 2D or 3D model, defined by the material construct (e.g. type of hydrogel) and within the well-plate or device which may govern cell population-population connectivity, Figure 1.

For some tissues, such as bone, cells will remodel the environment rapidly with no central need for precise cell-to-cell connectivity; for other, more complex tissues such as those of the nervous system, cellular architecture is of primary importance to the overall function of the tissue. For this reason, the long sought-after fabrication of whole organ systems remains challenging, yet there have been some major advances in bottom-up biofabrication approaches such as precise multi-cellular bioprinting, Figure 1(b), in addition to top-down use of decellularized matrixes to rebuild complex tissues.^14,15^ These approaches have clearly shown the advantages of 3D construction compared to the use of 2D tissue models, driving the regenerative medicine field by providing a more native-like cellular environment. Recent advances in 3D culture technology and tissue engineering have permitted the generation of 3D organoids that can partially recreate the anatomical structure, biological complexity and physiology of different tissues,^16^ whilst addressing the issue of limited cell availability apparent in other methodologies.^17,18^ In vitro stratified retinal organoids (RO), in particular, have been demonstrated to mimic the native retinal tissue spatiotemporal development in a way that cannot be observed in animal models.^19^ RO technology has revolutionized the field of ocular sciences, not only in providing advanced in vitro research models but also, enabling the generation of clinically relevant numbers of retinal cells for transplant therapies for the first time.^20^ Nonetheless, such methodologies usually are complicated, costly, time consuming and, particularly important in the case of RO generation, protocols need important standardisation of methods between labs.

**Figure 2.**
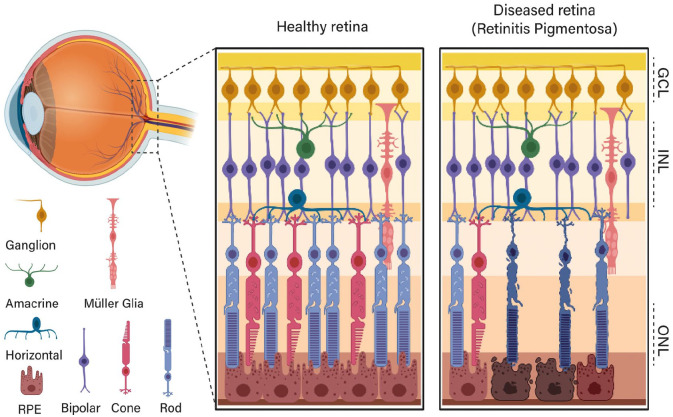
Schematic of a healthy versus diseased retina. The figure above shows a schematic of a healthy, functional retina. The light travels from the front of the eye across the first and transparent layers of the retina before reaching the photoreceptors at the back of it. The transduced electrical impulse travels from the photoreceptors towards the retinal ganglion cells and then to the brain. The figure elicits how retinal degenerative diseases, such as Retinitis Pigmentosa, damage the physiology of the retina firstly affecting the retinal pigmented epithelium (RPE) cells and the rod and cone photoreceptors, causing irreversible vision disfunctions in the patient. GCL: ganglion cell layer; INL: inner nuclear layer; ONL: outer nuclear layer. Created with BioRender.com.

A plethora of tissues have been engineered reaching clinical transplantation, such as skin,^21^ cornea,^22^ heart,^23^ bone^24^ and tracheal structures,^25^ however, the ability to create a functional retina in vitro *remains* a major challenge.^26^ The mammalian retina is a highly complex vascularized tissue containing seven primary cell types: rod and cone photoreceptors, amacrine cells, retinal ganglion cells (RGC), horizontal cells, bipolar cells and Müller glia cells,^27^ in addition to at least 60 functionally different cell types.^28^ Such cells are organised and divided into three main layers comprising the outer nuclear layer, formed by the photoreceptor outer segments containing the photosensitive pigments; the inner nuclear layer, mainly constituted by the cell bodies of bipolar, horizontal, amacrine cells and their fibres; and the ganglion cell layer, which contains ganglion cell bodies, whose axons constitute the optic nerve and send the visual information to the brain, Figure 2. These different cell types emerge from a common pool of multipotent neural progenitors cells which during embryonic development experience unidirectional changes in competence, producing differentiated cells in a precise spatiotemporal overlapping order, controlled by cell-intrinsic and extrinsic molecular mechanisms that determine retinal cell fate.^27,29^ Synaptic connections between the cells and their physiological needs must be perfectly orchestrated for the ‘*tissue*’ to be functional. Due to these limitations inherent to the tissue there is currently very limited literature on retinal tissue engineering.

Only very few studies have shown bioprinting of retinal cells.^30–32^ Importantly Masaeli et al.^31^ generated a functional RPE-photoreceptor system utilizing inkjet bioprinting technology using a carrier-free approach. Although results were promising, (Müller glia, ganglion, photoreceptors and RPE cells^30–32^) tissue engineering technologies are yet to show their capacity in printing all the different retinal cell types to develop a fully complete and functional in vitro retina.

### Microfluidic neural platforms

It is foreseen that some of the present limitations of 3D tissue engineered systems could be addressed using so-called ‘*microfluidic*’ *or ‘lab-on-chip’* platforms, Figure 1(c). Such systems allow the microscale positioning of single cells in a highly complex 2D system, breaking down the inaccessible complexity inherent to common 3D tissue engineering strategies. Microfluidics has the potential of quantifying biological processes at a single cell level and with high temporal resolution.^33^ These platforms are extremely versatile and have been widely applied to neuronal engineering but not often focussed on retinal tissue specifically. Being versatile in their design, microfluidic systems offer the potential to guide the connectivity of multiple populations,^34^ with microchannels designed to direct axonal connections only in a single direction as would be found in native tissues. Valves and pump systems can be incorporated to study different vascular strategies that allow functional maturation and long-term viability of the tissues, overcoming one of the prevalent issues 3D tissue engineering technologies currently present.

Strategies to model *retina-on-a-chip* have largely focussed on explanted tissues^35^ or simple multi-cellular systems not bespoke to retinal cell types,^36,37^ largely focussed on blood-retinal barrier parameter rather than engineering of functional retinal tissue per se. Much of this work is somewhat hampered by the lack of functionally active retinal cell lines, with only few well-characterised.^38^ Retinal organoids are known for the self-organisation of multiple cell types, organised into specific retinal architecture, albeit within a spheroid volume.^39^ The future of r*etina-on-a-chip* devices therefore could evolve from retinal *organoids-on-a-chip*, offering functional tissue investigation. Optogenetic therapies could certainly benefit from these in vitro devices – current in vitro analyses presenting immunohistochemical assessment of synaptic proteins expression together with voltage recording patch clamping^40,41^ or calcium imaging^39^ show promise, but are not entirely robust to fully understand the behaviour and functionality of engineered cells; advancing *retina-on-a-chip* technology would enable network functional assessment. In vitro models for the study of the retinal circuit and associated diseases will certainly extend our understanding of normal tissue function and sites of pathology, with such technology being central in the development of potential therapies.

### Ethico-economic reasons for tissue engineering

The increasing ethical and practical concerns regarding the use of animals for scientific research necessitates the development of novel in vitro models. Furthermore, the current need of complex biological and cell-based therapies, moving away from simple small-molecule drugs,^42^ need to be accompanied by more representative and clinically orientated models. The common issue for application of 2D cultures and in vivo animal models is their reliability, and bridging the gap between human and experimental animal models is difficult.^32^ The inability of current models to effectively mimic the in vivo human environment hinders research and the generation of novel clinical therapies.

Animal models fail to reliably recapitulate human physiology and pathophysiology, and therefore results from such experiments are often inconsistent and not directly translatable to human data, having to be extrapolated to predict the human scenario.^39,43–45^ Furthermore, in the case of the retina, none of the small animals used in the field are able to fully represent the human retinal system.^39^ The low throughput and the high cost associated with the use of animal models is one of the main reasons why the development of new drugs has become so inefficient, expensive and time consuming.^11,46^ Despite current technological developments and more efficient compound identification, 90% of compounds fail to progress through phase I clinical trials, resulting in colossal economic losses and delays in therapy development^47^ Conversely, although 2D models limit (primarily) ethical issues surrounding in vivo animal research, they fail to provide a realistic cellular microenvironment and therefore they cannot fully recapitulate cellular behaviours.^48^ More advanced in vitro human tissue models such as stem cell-derived 3D organoid systems, *organ-on-a-chip* or 3D bioprinted platforms may significantly improve accuracy, efficiency, reproducibility and therapeutic translatability of animal-free research, whilst also resolving the ethical concerns associated with animal testing.^32^ Representative and reliable human in vitro tissue models are needed to improve preclinical drug screening, and therefore the efficiency and success rate of clinical trials^42,44^ in an environmentally friendly and ethically conscious manner.^49^

This review brings together the diversity of approaches used for retinal tissue engineering, putting into context the related works on neural engineering more generally. With major developments in our understanding and technical abilities, the key challenges in engineering complex and functional tissue are now being approached, with future perspectives being proposed.

## Current clinical approaches to retinal pathology

In most retinal degenerative conditions, such as AMD and RP, RPE atrophy is associated with degeneration of the adjacent photoreceptors. Therefore, two main strategies have been followed for the regeneration of the retina and its function: restoration of photoreceptors or RPE cells (or their function *via* genetic modification). A variety of cell, gene and optogenetic approaches have been explored with efforts to bring them closer to clinical reality underway.

### Cell therapies

Replacement of lost retinal cells using stem, progenitor or mature neural retinal cells have been clinically explored, currently being the only feasible option to restore vision in patients presenting advanced states of retinal degeneration. Cell therapy approaches are broadly applicable to many inherited retinal degenerative diseases because they act independently of the specific genetic defect.^9^ Therefore, cell transplantation approaches for the replacement of lost photoreceptors or RPE cells is a major goal in the research community and has been investigated for the last three decades.^10,50^ Pioneering murine studies evidenced the potential of cell transplantation therapies demonstrating the survival of primary grafted photoreceptor cells into the murine subretinal space for up to 9 months.^51^ A major breakthrough was achieved by MacLaren et al., whereby the early postnatal period at the peak of rod photoreceptor genesis was identified as the optimal age of donor photoreceptors for transplantation, corresponding to post mitotic committed photoreceptors. This resulted in a higher survival and improved integration rates of the transplanted primary mouse rod photoreceptors, following successful and functional transplantation in mice, highlighting cell replacement therapy as a promising and viable approach to address retinal diseases.^52,53^ However, a problem concerning the scalability of cell manufacture was presented, with limited numbers of cells able to be expanded in vitro due to their tendency towards a postmitotic ontogenic state. Therefore, an expandable and renewable source of donor cells was required.

The generation and establishment of murine and human embryonic stem cells (ESC), and induced pluripotent stem cells (iPSC)^54–56^ provided a step-change in the field, allowing the unlimited expansion of donor cells being readily viable for clinical translation. Using a specific combination of factors, hESCs (human embryonic stem cells) can be directed to a retinal fate to generate retinal progenitors.^17^ Various groups have since studied the transplantation of derived rodent and human ESC and iPSC photoreceptors and RPE cells, reviewed elsewhere.^50,57^ Following the transplantation of photoreceptors, restoration of light responses to otherwise unresponsive animals has been demonstrated.^17^ The majority of the differentiated cells had, however, an inner retinal identity with only 12% expressing early photoreceptor markers and a very limited number of cells expressing typical markers of mature photoreceptors such as Recoverin or Rhodopsin.^17^ Longer cell culture times with the addition of retinoic acid (RA) and taurine, made it possible to increment (up to 10%) the number of opsins expressing photoreceptors.^18^ However, 2D culture systems remain quite limited in their ability to present a realistic biomimicry of the retinal niche environment.

Clinical trials focusing on cellular treatments for retinal diseases have shown encouraging results and demonstrated the safety involving cell transplant in humans.^58^ It is important to note that whilst RPE replacement may slow the degenerative process, it is likely that the restoration of vision will need replacement of photoreceptors as a ‘retinal sheet’, as an organised structure in which cells are delivered ready to be incorporated into the native tissue, or as a suspension of dissociated cells.^10,59^ Nevertheless, integration into the host retina and reconstruction of the neural circuitry and functionality are currently the major hurdles for successful cell transplantation.^10^

The most recent and ongoing trials can be classified into the transplantation of RPE cells as a suspension against senile macular degeneration, dry AMD and myopic macular degeneration^60–64^ transplantation of RPE cells as a monolayer,^65^ transplant of cells delivered primarily for cell rescue effects,^66,67^ foetal neural retinal cells (results not yet published), encapsulated cells secreting neuroprotective growth factors,^68^ and transplantation of retinal sheets.^69–71^ If found in earlier stages, an alternative method to cell transplant therapies is to cure or prevent disease by providing a gene with therapeutic action.

### Gene therapies

Retinal diseases associated with loss or gain of function mutations can be treated with gene therapies by correcting the causative gene mutations *via* gene supplementation or endogenous gene suppression.^72–74^ The therapeutic gene can be delivered by a variety of viral vectors injected directly into the eye. Since the successful results of the first approved ocular gene therapy, a gene supplementation of RPE65 in patients with Leber congenital amaurosis,^72–74^ many more gene therapies are currently in the clinical phase of development for the treatment of inherited retinal dystrophies including RP,^75–78^ age-related macular^79^ or Leber hereditary optic neuropathy,^80^ among others.^81,82^ Although gene therapies are a promising clinical tool, there are limitations regarding the diseases they can be used to treat, with outcomes dependent upon the stage and progression of the disease. Gene therapy strategies that restore function within the retina, are limited only to those patients still presenting functional photoreceptors.^6^ In such cases, intervention is required at early-stage disease progression when the retinal structure is undamaged.

### Optogenetics

In advanced stages of retinal degeneration there is a low probability of clinical benefit from gene replacement therapies since most of the photoreceptors are absent. Optogenetic techniques are approaches which change the neural activity in the retina, by generating ‘*new photoreceptors*’ from surviving inner bipolar or ganglion retinal cells. Despite the loss of photoreceptors, in most cases of advanced disease states 88% of the retina’s inner nuclear layer and 48% of the ganglion cell layer remain viable, maintaining the ability to send visual information to the brain.^83–85^ Genetic introduction of light-sensitive proteins (microbial opsins or chemical photo-switches), can photosensitise non-photosensitive cells and thus, restore visual function in late stages of degeneration.^86,87^ Retinal remodelling is believed to be caused by deafferentation, and consequently it has been proposed that optogenetic-driven light sensitivity restoration in inner retinal neurons (and re-activation of retinal activity as suggested for gene therapies) could also prevent or delay the remodelling processes.^88^ Furthermore, the introduction of optogenetic sensors in iPSC-derived photoreceptor precursors could bypass the need for a functional maturation of transplanted cells, independent of the formation of outer segments and the presence of RPE.^10^

Optogenetic therapies are currently not targeted to specific genetic mutations, and therefore, unlike gene therapies, could be used to treat a wide variety of inherited retinal degenerative diseases.^9^ However, the degree of restored vision driven by optogenetics approaches is dependent on the targeted cell type and the state of retinal degeneration.^9^ One of the biggest limitations these techniques have is the lack of adaptation of the engineered light-sensitive cells. Current methodologies do not provide sufficient data to unequivocally evaluate the engineered photosensitive capacity of the cells after transduction, and due to the lack of current clinical trials in humans, it is not yet possible to know how the regained visual capacities will be interpreted by the patients’ brain. Therefore, a major obstacle to clinical translation of this technology is the safety and the efficacy concerns related to the use of microbial opsins, in addition to the intensities required for their activation. Further, engineering of new light-sensors together with improved integration of transplanted cells may represent substantial advancements for the efficacy of cell replacement using optogenetic sensor-expressing iPSC-derived cells.^10,89^

## Dimensionality: 2D versus 3D

2D in vitro cell cultures have served as strong tools for biomedical research since the establishment of the technique in the early 1900s.^90^ They have provided tremendous insights into a wide range of cellular and physiological interactions within the human body.^91^ To date, numerous methods have been developed to generate complex 2D patterns and gradients of physical and biochemical cues, in order to imitate native biological environments.^91,92^ For example, 2D neural culture studies are commonplace, particularly in the areas of axon/dendrite growth, neuronal survival and synapse formation.^93^ Despite these advances, the number of cells such systems can generate is limited and therefore, classic 2D culture models fail to recapitulate the complex architectures of heterogeneous or vascularized tissues and organs. This is in part is due to the cultures lacking in the complex cellular, topographical, biochemical and mechanical stimuli found within the natural 3D organism,^48^ affecting the quality and accuracy of the generated data as a representation of the organism.^94,95^ Furthermore, researchers’ perceptions are changing towards the idea of understanding cell networks and their interactions, rather than individual cells, as the functional unit of study; with different populations and (multi-)co-cultures of cells forming functional tissues. Hence, current neuroscience studies predominantly focus on neuronal circuits and how the information is processed and transmitted, as opposed to single neurons.^96^

3D models that re-establish in vivo-like cell-cell and cell-ECM physiological microenvironments aim for the recapitulation of complex cellular architectures found in tissues and organs for the generation of transplantable artificial organs, research tools for modelling diseases or developing new therapeutic strategies.^91^ In such systems biomaterials are necessary for the integration of cells into 3D tissues. Interactions between cells and biomaterials under optimal conditions permits the progression from individual cell cultures towards the generation of functional tissues including more than one cell type. Modern fabrication technologies and new biomaterials, together with the increasing knowledge in cell physiology and behaviour have enabled the adaptation of 2D methods. It is now possible to generate novel systems that can accurately mimic the native 3D heterogeneous cellular environments of endogenous tissues and organs.^91,97^ A wide variety of cell types can be supported in vitro permitting 3D organs to be engineered in the laboratory, for example, *via* 3D bioprinting or ‘*bioplotting*’.^49^

In addition to scaffold architecture and its physio-mechanical properties, cells also respond to a variety of stimuli, including surface chemical and topographical features, mechanical and electrical activation.^98,99^ Electrical stimulation of electroactive neural cells/tissue has been shown to enhance culture growth by promoting adhesion, alignment and cell maturation. As a result, electrical activity has a notable role in a modulating neural behaviour and influence the development and regenerative processes of the central nervous system (CNS).^100^ It has also been demonstrated that exogenous electrical stimulation affects neural cell parameters such as cell migration,^101,102^ neurite outgrowth,^103^ and/or maturation.^104^ Biochemical signalling cues (e.g. peptides, growth factors, cytokines, or other signalling molecules) are all inherently located within the 3D matrix with very specific spatio-temporal concentration dynamics. Elements of the extracellular environment guide cellular behaviour, determining cell fate, mimicking the cellular architectures and functions found in tissues in vivo.^48,91^ Such physical and chemical elements are often presented as gradients in the in vivo environment, which are established in organs and tissue morphogen gradients during organogenesis.^105^ Taking this aspect into the design of 3D in vitro constructs, gradients of physical factors, such as mechanical stiffness, chemical and signalling factors, have been shown to have great impact on biological processes^97^ including axon guidance.^106^ The implementation of methods that can replicate such heterogeneous physiological gradients in engineered tissues are required for the development of functional and realistic models.^92,97^

**Figure 3.**
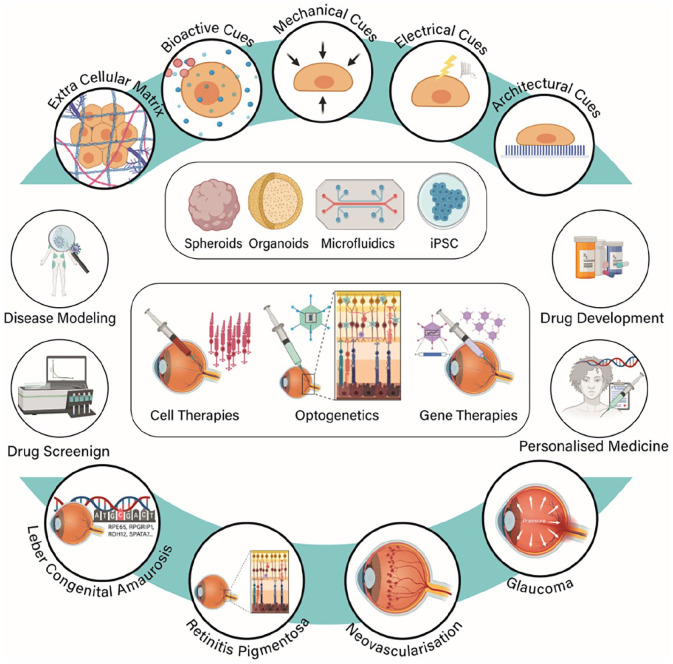
Schematic illustration of tissue engineering strategies necessary to model injuries and diseases of the retina and the complex architecture of this tissue. Biophysical and biochemical cues must be engineered into a 3D matrix through different technologies and then integrated with different cell types. Using these 3D in vitro models, it would be possible to investigate underlying pathophysiological mechanisms and future therapeutic approaches, screen drug candidates and develop platforms for personalised medicine. Created with BioRender.com.

In the CNS, neurotrophins modulate the cellular environment to accelerate neuronal growth and functional recovery following, for example, CNS injury.^48^ Neurotrophic factors have been used as nerve guides following peripheral nerve damage using for instance, bioactive and biodegradable chitosan scaffolds loaded with Neurotrophin3 (NT3) to enable robust neural regeneration accompanied by motor and sensory functional recovery.^107^ In retinal tissue, several growth factors, including insulin-like growth factor (IGF)-binding proteins, connective tissue growth factor (CTGF, also known as CCN2) or leukaemia inhibitory factor (LIF), among others have been identified to have neuroprotective effects, thus increasing photoreceptor survival.^81,108^ Interestingly, it has been demonstrated that Müller glia cell conditioning media supports the culture of photoreceptors, which usually die after one or two days ex vivo, due to the synthesis of a variety of neurotrophic factors such as brain-derived neurotrophic factor (BDNF), ciliary neurotrophic factor (CNTF), basic fibroblast growth factor (bFGF), pigment epithelium derived factor (PEDF), or glial derived neurotrophic factor (GDNF) to name but a few.^109^ Similarly, human vascular endothelial growth factor (VEGF) protein is a potent endothelial factor which promotes angiogenesis and its optimal levels are essential for maintaining choriocapillaris and choroidal vessels in vivo.^110^ Furthermore, it also has neuroprotective effects for neural retina and acts as an anti-apoptotic agent for retinal neurons.^111,112^ Application of such knowledge into the development of retinal 3D models and *retina-on-chip* devices would support and enhance the generation of in vitro functional retinal tissues. Identification and recapitulation of the aforementioned parameters are essential for the generation of neural tissues with maximal authenticity, Figure 3.^12^

### Tissue engineering of the delicate neuro niche

**Figure 4.**
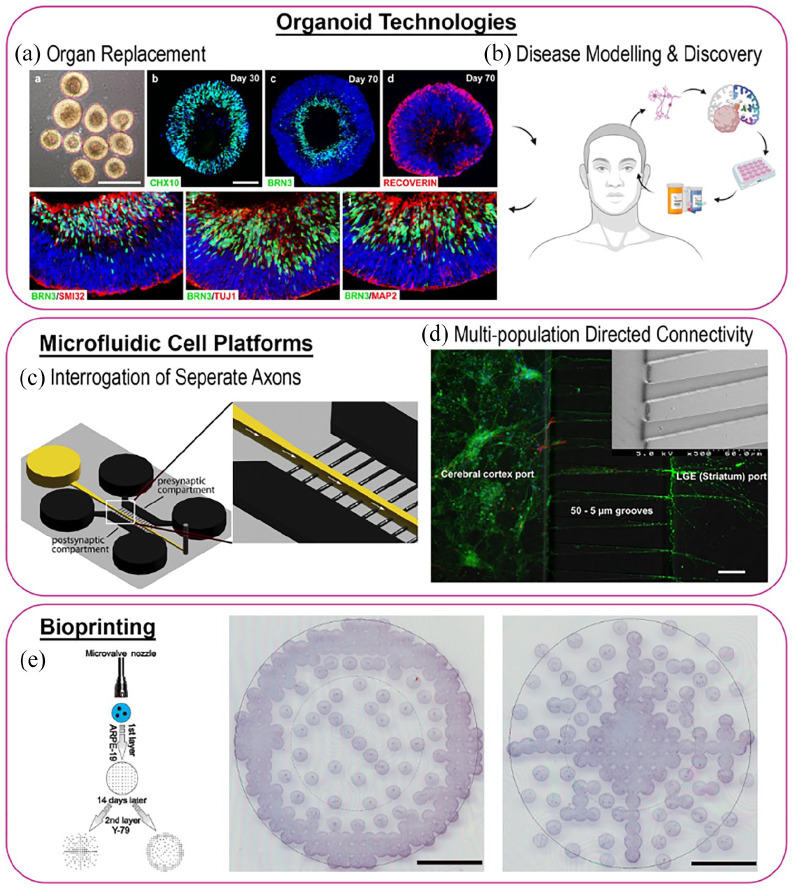
Retinal tissue engineering and biofabrication approaches. Retinal organoids present an excellent tool for the study of organ replacement, drug development and personalise medicine, for the study of retinal diseases and retinal regeneration. It is the only approach that can provide with the relevant number of cells needed in the clinic. Nonetheless, retinal organoids 3D organised structure makes them inaccessible and presents difficulties for analysis. Microfluidic cell platforms combine the cell compartmentalization capabilities, between 2D and 3D, with a unique analysis performance that can examine cell-cell interactions. Bioprinting technologies offers an extremely precise cell allocation capabilities to generate complex 3D structures 32, that can, in some cases, recapitulate the native in vivo tissues. Reproduction of images are under copyright: (a) Creative commons attribution 4.0 international license (https://creativecommons.org/licenses/by/4.0/legalcode), Fligor et al.^179^ (c) CellPress, Taylor et al.^201^ (d) creative commons attribution 3.0 licence (https://creativecommons.org/licenses/by/3.0/legalcode), Kamudzandu et al.^34^ (e) John Wiley and Sons, Shi et al.^32^ Created with BioRender.com.

Different tissue engineering strategies can be used to create complex cellular environments using biomaterials. 3D printing has quickly become an attractive method to rapidly fabricate complex architectures using a top-down approach.^91^ This automatized and reproducible method allows for the precise control of material properties such as pore size and surface area. Furthermore, 3D printing allows the *layer-by-layer* fabrication of complicated structures that are difficult to manufacture using traditional approaches such as subtractive or formative manufacturing.^113^ As a branch of 3D printing, 3D bioprinting utilises encapsulated cells in prepolymer suspension or hydrogel matrices as the ink, referred to as bioink. Precise and controlled deposition of the bioink permits the building of functionally complex 3D constructs composed of different cell types that better imitate the intricacies of the natural physiological environment of tissues and organs in comparison to 2D models, Figure 4.^49,114^ Neurons are extremely sensitive to the surrounding microenvironment, and thus stresses generated during printing cause major concerns regarding cell viability and deformation.^12^ There are, however, examples of neural cell bioprinting with a good degree of viability after taking into consideration factors of the cell and bioink processing.^115,116^ In addition, patterning neuronal tissues that are functional post-printing remains a challenge.^117^ The following sections will cover issues more generally of neural cells, with a separate focussed section for retinal-specific cell types.

An early study by Xu et al.^118^ demonstrated the effects of thermal inkjet printing on neural cell viability (specifically rat embryonic motor neurons in a phosphate-buffered saline solution) and demonstrated that neural cells are printable. In addition, they investigated whether printed neuronal cells (neural crest-derived neuroblastoma line and an embryonic rat brain cell line) could maintain functionality; it was demonstrated that 75 % of the printed cells were viable following 8 days of culture, even exhibiting electrophysiological properties.^118^ Furthermore, multi-layered neural sheets were constructed in which neurite outgrowth was observed.^119^ These results demonstrated the feasibility of printing neural cells *via* inkjet printing, showing the potential of constructing neural tissues using this technology.^12^

In 2009, Lee et al.^120^ presented the direct inkjet printing of 3D multi-layered collagen gels with well patterned rat embryonic astrocytes and neurons and revealed that cell viability was not greatly influenced by the printing process. In fact, they demonstrated neurite outgrowth and neural connectivity in the resulting 3D construct. More recently Lozano et al.^121^ used an extrusion 3D bioprinter to print a brain-like structure consisting of two layers of encapsulated primary cortical neurons within a peptide-modified hydrogel. They demonstrated successful encapsulation, survival and networking of the cells in the 3D printed constructs, with axons penetrating into the acellular layer. Their results demonstrate the versatility of bioprinting to control cell and ECM organisation for the construction of complex and viable 3D cell-containing constructs.^48^ Furthermore, their methodology replicated more precisely the 3D in vivo brain environments compared to previous models, providing a useful tool for the study of cell behaviour, brain injuries and neurodegenerative diseases or drug testing.^121^

Regarding the peripheral nervous system, Owens et al.^122^ developed a proof-of-concept model and completed functional testing for a new type of nerve graft. They bioprinted a fully biological graft composed exclusively of cells (using Schwann and mouse bone marrow stem cells) and cell secreted materials. Similarly, England et al.^123^ encapsulated Schwann cells within a fibrin-factor XIII-hyaluronate composite. The scaffolds mimicked the natural fibrin clot that forms between injured nerve ends. Furthermore, the aligned, encapsulated Schwann cells may provide natural guidance of neurite growth enhancing current methods for peripheral nerve regeneration *via* the use of acellular printed nerve guides used in the clinic such as NeuroGen^®^, Neurolac^®^ and Neurotube^®^.^113^ 3D printing for peripheral nerve regeneration could provide an enduring area of future research. The inclusion of matrices/bioinks, scaffolds, neurotropic factors and transplanted cells could achieve functional regeneration over distances larger than 18 mm of nerve gap.^113^

Due to the lack of accessibility to human neural tissues, bioprinting studies using human cells are scarce. Gu et al.^124^ printed a porous 3D scaffold incorporating human neural stem cells with a novel and clinically relevant polysaccharide-based bioink that allowed the proliferation and differentiation of the cells. They showed functional maturation of the neurons which formed synaptic connections and established spontaneous network activities. In the same year, a novel bioacoustic levitation assembly approach was developed to engineer 3D brain-like constructs.^125^ The levitated human neural progenitor cells were immobilized in a fibrin hydrogel construct and differentiated into neural cells in the 3D microenvironment to form both inter-and intra-layer neural connections. Bioacoustic levitation assembly provides a simple, rapid, and biocompatible method to bioengineer multilayer tissue constructs for a wide array of applications including neuroscience, cardiovascular and cancer biology.^117^

Despite the great potential and recent advances of bioprinting, there are several limitations that still need to be addressed; these include the need to improve the overall resolution; formulating cell/printing permissive bioinks with fully defined components; obtain good spatiotemporal control over signalling gradients and, supply sufficient nutrients and oxygen.^12^ Bioprinting has already shown its capacities for the precise spatial positioning of cells (Figures 1 and 4) and it’s feasibility of fabricating complex heterogeneous tissue constructs containing multiple cell types.^13^ Studies investigating the bioprinting of neural, and more specifically retinal tissues, are currently limited, with only a few types of cells successfully printed^30–32,126–128^ but with no current demonstration of the range of cells needed to engineer a functional retinal tissue. Significant developmental effort and capital investment is required to realise an implantable 3D printed retina in the future.^26^ Bioprinting alone or in combination with the other technologies, will likely achieve clinical success initially with less complex tissues, such as bone or skin, before potentially achieving fully functional complex organs and tissues such the retina. Scaffolds capable of both differentiating stem cells whilst providing microenvironmental cues that allow the maturation of these cells into adult tissue, could develop organs on demand in vitro in the future.^91^

### Materials for retinal tissue engineering

Although the in vitro engineering of a full functional retina is yet to be achieved, with limited development of biomaterials specifically to retinal tissue and no commercial products currently available,^1^ important progress has been made towards engineered RPE and the neural retina. Researchers have used biomaterials based on Bruch’s and amniotic membranes,^129–131^ alginate,^132,133^ silk,^134,135^ cellulose,^136–139^ and hyaluronic acid^140–142^ to create scaffolds to support retinal tissue development or to act as Bruch’s membrane substitutes or cell delivery systems. Comparisons of key studies, the different methodologies and materials used, and the advantages and limitations of such studies has been reviewed elsewhere.^1^

Synthetic and natural polymers have also been investigated for retinal tissue engineering. Scaffolds with well-defined requirements, such as biocompatibility, ultra-fine structure and appropriate physico-mechanical properties adequate for both manipulation and implantation, have been introduced to guide retinal repair.^31^ Polymers such as polylactic acid, polylactic-co-glycolic acid, polyglycerol-sebacate, poly(e-caprolactone) and poly(ethylene glycol) diacrylate, amongst others, have been studied to guide the delivery of photoreceptors or RPE cells into the subretinal space,^143^ as a substrate for engineering the RGC layer,^144,145^ or as a Bruch’s membrane substitute.^146,147^ In addition, incorporation of nanowires (2.5/27 μm) projecting from a polymer base can promote the migration and integration of the grafted cells into the host retina.^148^ In general, such studies have shown that the use of polymers presents different advantages such as higher proliferation and differentiation, increased cell survival and improvement to the number of successfully delivered cells to the subretinal space or induction of retinal progenitor cells differentiation and generation of functional neurons.

It has been proposed that some biomaterials may enhance retinal tissue formation in addition to facilitating delivery/integration in vivo.^140^ The bolus injection of cells to the subretinal space, the current gold standard technique for cell delivery in retinal therapies, results in disorganized and poorly localized grafts.^149^ Such limitations inherent to the transplantation procedure, result in low rates of cell survival due to poor donor cell integration and injection reflux.^149^ Injectable hydrogel cell delivery systems have been studied as they may decrease donor cell death as a result of the injection reflux.^140^ Such injectable materials allow for provision of a scaffold without the need for large incisions to be made in the retina.^1^ Nonetheless, a single polymer is unlikely to meet the needs of both RPE and photoreceptor replacement therapy.^143^ Since RPE cells tend not to attach/survive on an aged Bruch’s membrane, a (polymeric) support material for RPE replacement is essential for localisation of cells, their survival, and directed functional differentiation; however, a permanent barrier in the sub-retinal space would compromise photoreceptor transplant functionality. Unfortunately, limitations such as the cell delivery method, the integration of grafted cells and their incomplete differentiation currently hinder the success of cell-based therapies.^31,150^

Transplantation of donor retinal cell sheets has been shown to be a more organised and controlled approach whereby cells are properly oriented and at the same differentiation stage.^143^ This approach does not rely on polymeric scaffolds or hydrogels for cell delivery, it consists of harvesting cultured cells as intact sheets along with their ECM and combines them to form tissue-like structures.^31^ A variety of studies have reported the successful generation of transplantable RPE cell sheets.^151,152^ Retinal sheets can morphologically repair an area of a degenerated retina, and there is evidence to suggest that transplants form synaptic connections with the host and restore visual responses in animal models.^153–156^ In 2014, Assawachananof et al. reported encouraging morphological and functional results in animal models after transplantation of full-thickness RO-derived sheets for advanced retinal degenerative diseases, through *proof-of-concept*; this has been more recently addressed by MacLelland et al.^157^ Similarly, a recent ‘wine glass’ scaffold design has been shown to promote efficient capture of human pluripotent stem-cell-derived photoreceptor cell bodies and guidance of basal axon extensions, achieving a uniform level of organization and polarization, not possible with bolus injections or previously described scaffolds.^158^

### Retinal tissue construct engineering

Although bioprinting of a wide variety of neural cells has been demonstrated, retinal cells have received much less attention compared to, for example, peripheral nerve cells. This is likely due to the availability of retinal cell lines and/or difficulties in separation of specific sub-cellular types from a primary source. Bioprinting of retinal tissue presents some attractive characteristics, these include: *(a) High throughput applications*: the native tissue structure and cellular interactions can be rapidly reproduced to investigate cell functions, whereas manual cell seeding is time consuming with low precision; and *(b) Accuracy and efficiency*: the process is well controlled to maximally reduce manpower and exclude man made errors.

Lorber et al.^30^ were the first to demonstrate that piezoelectric inkjet printing technology could be used to print retinal cells (murine adult ganglion cells and glial cells) for the development of a tissue graft. Piezoelectric printing has been less commonly used to print cells due to concerns regarding cell membrane integrity.^118,119^ However, Lorber et al.^30^ did not observe significant differences in cell viability and neurite outgrowth between the printed and non-printed (control) cells, suggesting that the cells are not adversely affected by the printing process. Interestingly, neurite outgrowth was increased significantly when the glia cells were used as a substrate for printed and control RGC, probably due to the neurotrophic factors they produce.

In 2016, Kador et al.^126^ printed retinal ganglion cells onto hydrogel matrices embedded electrospun scaffolds to mimic the directionality of the nerve fibre layer in vivo. Whilst retinal ganglion cell survival was not compromised, electrophysiological function of the cells was retained, as printed cells required higher injected current to elicit action potentials. Cell density was 70 times less than in an in vivo retina, thus illuminating that further advances in bioprinting technologies are needed to model 3D electrically functional retinal systems.

Laude and colleagues used a microvalve-based bioprinting technique to generate two distinctive retinoblastoma (Y79) cell-seeding patterns on top of a previously bioprinted monolayer of RPE (ARPE-19) cells.^32,127^ The study demonstrated that the technique was able to precisely and effectively deliver the cells to form an in vitro retinal tissue model *via* 3D cell bioprinting. The resulting model could simulate key aspects of native/diseased tissue compositions in terms of cell distribution and cell density, not seen in the manually seeded controls. Nonetheless, Laude’s in vitro model used retinoblastoma tumour cells (that have been only shown to differentiate into photoreceptor, neuronal and glial cells) not differentiated into any specific cell type, which is likely to limit its usage. Bioprinting of retinal progenitor and RPE cells within hyaluronic acid hydrogel have been shown to be a promising approach for the 3D bioprinting of a retina.^128^ Chemical alteration of the hyaluronic acid hydrogels to match the compressive modulus of the native retina was demonstrated to support cells functions and allowed the co-differentiation of the retinal progenitor cells into photoreceptors with the support of the RPE cells.

More recently, Masalli et al.^31^ presented a novel carrier-free bioprinting method to generate an in vitro retina, showing for the first time that both RPE and photoreceptors can be bioprinted and express essential transcription factors. A unique inkjet bioprinting system was used for the precise deposition of the cells following a predefined arrangement to create complex double-cell sheets. A photoreceptor cells layer was bioprinted on top of a RPE cell layer, previously bioprinted over a thin layer of gelatin methacrylate (GelMa) coating used to mimic the Bruch’s membrane. They demonstrated that the GelMa Bruch-like membrane was essential in providing mechanical support for the RPE cells, recapitulating the native RPE microenvironment, and providing enriched ECM conditions for RPE cell culture, and likely facilitating cellular maturation after bioprinting. Both cell types were well positioned in a layered structure and expressed their structural markers in a in vivo-like manner. Electron microscopy images showed polarized RPE sheet with several features of RPE morphology, including numerous dense bodies in the cytoplasm and numerous microvilli. Importantly, 24 h after bioprinting the photoreceptors, the internal structure of the phagocytized photoreceptors outer segments could be observed in the RPE cells, a characteristic that has not been addressed in previous bioprinting studies. This data indicated that the bioprinting process reliably and robustly produces a GelMa/RPE/Photoreceptor complex with adequate functionality, protein expression and cell densities comparable with that of the native retina.^31^ Furthermore, they also showed the capability of the inkjet bioprinting technique to spread mature and differentiated photoreceptors over a large surface (up to 1 cm^2^), resulting in photoreceptors sheets from mature and freshly isolated cells. Although this study is a great step forward into the development of an in vitro retina using 3D printing techniques, and has reached a reasonable level of tissue complexity, it is still far from mirroring the specific spatial arrangements of the intricate circuits and multiple cell types that comprise the native retinal tissue (Figure 2). Furthermore, electrophysiological functionality of the bioprinted photoreceptors to receive, transform and transmit the stimuli is yet to be fully evaluated. In general, the findings from these studies need to be translated to all the cell types of the human retina, to progress with advanced models and therapeutic delivery. Specifically, aspects such as cell density, spatial and functional integration of the cells and long-term cell survival of the bioprinted constructs will need to be addressed.^26^

In order to generate a functional 3D retina, bioprinted cells must be able to correctly connect with each other to establish horizontal and vertical networks between the different layers to ensure appropriate physiological function and transmission of the visual information in the printed construct.^26^ An analytical method to comprehensively verify the functionality of the biofabricated constructs is yet to be developed, and currently used methods (e.g. immunohistochemistry or patch clamping voltage recording) are only able to suggest that the differentiated cells have synaptic machinery capable of forming functional synapses. It is crucial that next generation scaffolds incorporate electronic sensors to monitor the bioactivity of the cells within these 3D microenvironments in real time and be able to respond accordingly.

A comprehensive review of the different 3D biofabrication strategies for tissue engineering comprising the different materials and techniques in use, their challenges and future perspectives can be found elsewhere^91,159,160^ including those focused on neural tissues.^12,48^ Lorber et al. review the advances in retinal tissue bioprinting, its challenges and limitations. An excellent, complete, and detailed review of the application of biomaterials to tissue engineering the neural retina and RPE, including past and recent clinical trials, their methodologies and results has been presented by Hunt et al.^1^

It is noteworthy that, regardless of the increment in the tissue complexity and function, 3D tissue engineering is still very limited by the number of cells that can be generated in vitro and are still far from reaching in vivo numbers. Conversely, organoid technology can mitigate some of the limitations of 2D and 3D bottom-up tissue engineering systems. Organoids endogenously recapitulate the architecture and cellular organisation of native tissues, not only by reaching higher and more natural complexities compared to 3D tissue engineering techniques, but also allowing for the generation of clinically relevant cell numbers (and ratios of differing types). Research into organoid technologies is driving a rapid development in research models and translation of clinical therapies.

## Organoid technologies

Although the term ‘organoid’ has been extensively used in literature and sometimes taken to refer to embryonic bodies (their methodological predecessor) or spheroids, organoids are more complex and present unique characteristics that makes them one of the key emerging fields of modern and future regenerative medicine. Lancaster and Knoblich (2014) defined organoids as a ‘. . .*collection of organ-specific cell types that develops from stem cells or organ progenitors and self-organizes through cell sorting and spatially restricted lineage commitment in a manner similar to* in vivo’. In contrast to the cellular aggregates presented in spheroids or embryonic bodies, organoids can recapitulate some specific functions of organs such as excretion of determined hormones (e.g. thyroid organoid), filtration (e.g. renal organoid) or neural activity (e.g. brain organoid).

Organoids are typically grown ex vivo from pluripotent stem cells or isolated progenitor cells, in gels or media containing ECM components.^19^ Under these conditions, cells proliferate, self-organize, and differentiate to form isolated functional units that resemble the basic organ structure and function. Whereas in other culture methods extrinsic factors can be supplemented into media to cue cell differentiation, organoid technology relies on the self-organisation of cells and their intrinsic development signals to form complex cellular architectures. The lack of extrinsic factors during early stages of differentiation, the 3D tissue organization and contact with supporting hydrogels such as Matrigel^®^, may contribute to the creation of a microenvironment reminiscent of that found in vivo. Cell signalling drives the activation of innate developmental programmes resulting in the generation of stratified structures which closely resemble the spatiotemporal development in vivo.

Organoids have enormous potential to model development and disease, as a tool for drug testing, and as a therapeutic approach. Future efforts will no doubt bring them closer to reaching this potential. Generation of 3D structures recapitulating the brain tissue organization have been used extensively by investigators in the past several years.^19^ Pioneering work from the lab of Yoshiki Sasai showed the self-organized development of apico-basally polarized cortical tissues in embryonic bodies from murine and human ESCs using an efficient 3D aggregation culture. Furthermore, the generated cortical neurons were functional, transplantable, and capable of forming proper long-range connections in vivo and in vitro. Importantly, the generated tissues replicated spatiotemporal aspects of early cortico-genesis.^161,162^ Today it is possible to generate a wide variety of organoids such as intestine,^163^ liver,^164^ or kidney.^165^ A comprehensive review comprising of the history and development of organoids as well as an in-depth analysis of its advances and different types of organoids has been previously published.^19^

### Retinal organoids

In the last decade, ground-breaking advances in three-dimensional cell culture^41,166–168^ have enabled researchers to grow stem cell–derived optic cup-like structures, generating retinal-like stratified neuroepithelia and RPE.^9^ Since then, the capability to generate ESC or iPSC derived retinal cells has significantly advanced and it is now possible to generate clinically relevant and adequate numbers of cells for use in cell replacement therapies, which was not possible using 2D culture systems.^17,18,52,169^ RO technology provides a system which not only replicates, in a very accurate fashion in vitro, the species-dependent spatiotemporal in vivo retinogenesis, but also the self-assembling organisation and the natural cell-cell signalling results in the acquisition of a retina-like layered vesicle with morphology and characteristics of a functional retina.

**Figure 5.**
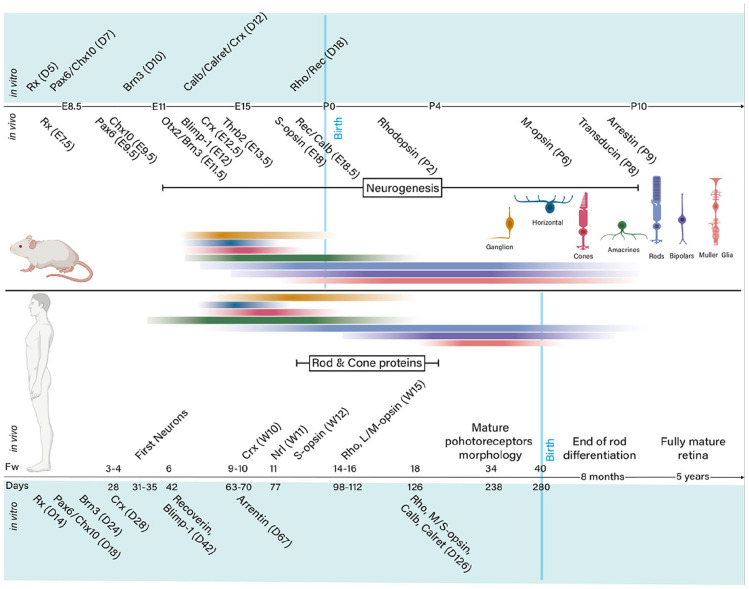
Comparison of mouse and human in vivo versus in vitro retinal development. Schematic representation of events in mouse and human retina development observed in vivo or in in vitro retinal organoids based on marker expression mainly through immunohistochemistry. Although human in vivo retinal development data is less precise, and literature detailing the process is scarce, a high conservation of the genes involved in retinal development exists across species. In the mouse, retinal development is well studied, and therefore, multiple markers can be used as a reference when analysing the resemblance of retinal organoid development to the in vivo situation. Rho, rhodopsin; Rec, recoverin; Calb, calbindin. Data collected from a range of experimental^166,168,173,174,228–231^ and specialised reviews.^20,89,232–234^ Created with BioRender.com.

**Figure 6.**
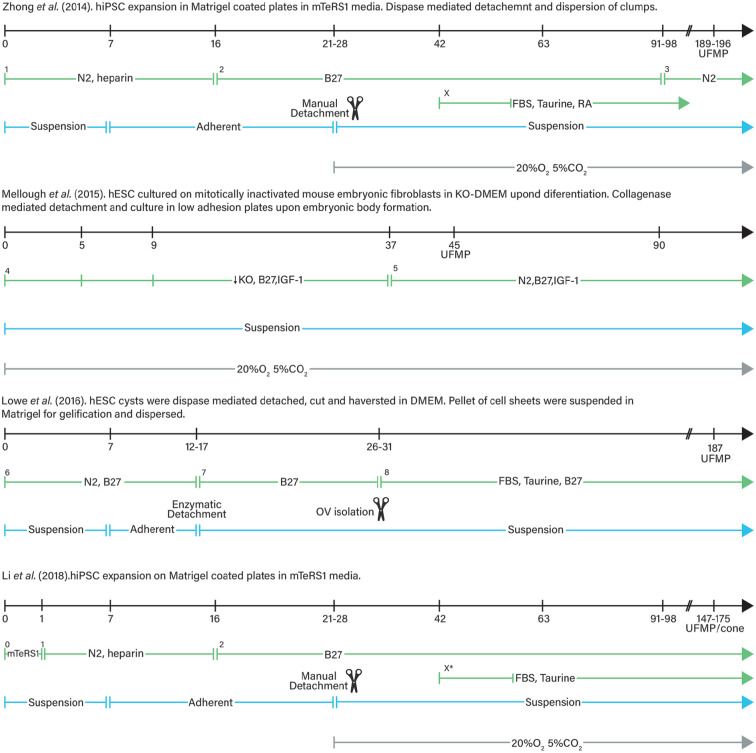
Comparison between different retinal organoid culture protocols followed by Zhong et al.^41^, Mellough et al.^170^, Lowe et al.^171^ and Li et al.^172^. Variation in the culture conditions is important as it is the different time at which retinal organoids mature and show ultrastructural features of mature photoreceptors (UFMP). 0. In mTeSR1 medium + 10 mM Blebbistatin (stem cells maintaining conditions); 1. Gradual replacement of mTeSR1 medium + 10 mM Blebbistatin with NIM = DMEM/F12, 1% N2,1X NEAA, 2 μg/mL heparin. Gradient mTeSR1 and NIM change from 3:1, and 1:1 ratio, to total NIM in the first three days of differentiation; 2. In RDM = DMEM/F12 3:1, 2%B27, 1X NEAA, 1% penicillin-streptomycin, 1% antimycotic; 3. In RDM = DMEM/F12 3:1, N2, 1X NEAA, 1% penicillinstreptomycin, 1% antimycotic; X. For long term culture RDM was added with 10% FBS, 100mM Taurine, 2mMGlutaMAX and 1 μM all trans RA (reduced to 0.5 μM by D112) from D42; 4. In KO-DMEM, 1mM Lglutamine, 100 mM NEAA, 1% penicillin-streptomycin and 8 ng/ml bFGF and B27 1:50 with reducing concentrations of KO serum (20,15 and 10 % until D5, D9 and D37 respectively and 5 ng/ml IGF-1; 5. In KO serum free DMEM, 1 mM L-glutamine, 100 mM NEAA, 1% penicillin-streptomycin and 8 ng/ml bFGF and B27 1:50 with of N2 (1:100) and 10 ng/ml IGF-1; 6. In N2B27 medium = DMEM/F12+Glutamax : Neurobasal medium 1:1, 0.5X B27, 0.5X N2, 0.1 mM B-mercaptoethanol, 2 mM Glutamax; 7. In DMEM/F12 3:1, 2% B27, 1X NEAA; 8. In DMEM/F12 3:1, 2% B27, 10% FBS, 100 μM taurine, 1X NEAA, 2 mM Gutamax; X*. Addition of 10% FBS, 100 mM Taurine, 2 mM Glutamax and 1 μM all trans RA. UFMP: ultrastructural features of mature photoreceptors; NIM: neural induction medium; NEAA: non-essential amino-acids; RMD: retinal differentiation medium; KO: konock-out; RA: retinoic acid; FBS: foetal bovine serum; IGF-1: insulin growth factor 1.

In vitro culture of 3D murine ROs (Retinal Organoids) was first demonstrated in 2011, a milestone in the development of structurally defined cell systems. Eiraku et al.^166^ were the first to show that mouse ESCs can self-organise in vitro into fully stratified and organised structures of the developed eye, in a spatiotemporally regulated manner, thus mimicking in vivo murine development (Figure 5). Later studies focused on the generation of human ROs^41,170–172^ following different differentiation protocols (summarised in Figure 6). Such studies demonstrated, as previously seen with mice, that in vitro multipotent human retinal progenitor cells differentiate in an ordered in vivo-like fashion in which RGC are generated first.^41^ When comparing the generation of the hESC-derived ROs with mESC, the following observations can be raised. Due to the recapitulation of the species spatio-temporal development, formation of mature hESC-derived RO takes much longer (~200-300 days) and results in a considerably larger structure compared to mESC (~25-30 days). Selection of the cell type and source is heavily reliant on characteristics required, budget and time considerations of the experiments to be conducted, and not least the link between human-relevant experimentation.

More recent studies have focussed their attention on the increment of the production of cone versus rod photoreceptor cells. The generation of cone rich organoids has a great importance as normal rod rich organoids do not represent a real advantage for cell replacement therapies since human daily vision is dependent on cone function. Therefore, studies to understand the mechanisms and factors underlying cone differentiation are necessary.^172^ Modification of initial protocols^166,173,174^ have resulted in the generation of a significant proportion of cone photoreceptors with high efficiently.^175^ This has permitted the isolation of large numbers of purified cones for transplantation into a model of end-stage degeneration for the first time.

In addition to cell replacement applications, organoids have the potential to drastically advance our understanding of biological systems, even those with high complexity. There are a number of examples where fundamental research has been carried out using organoids for a variety of proposes such as the study of pathogenic gene mutations and molecular mechanisms in retinal diseases such as RP;^176^ as a platform for drugs screening and personalised medicine;^177^ novel RGC neuroprotective factors;^178^ RGC organization and neurite outgrowth;^179,180^ or investigation of the role of ECM components during human retinogenesis.^181^ For example, the successful isolation of RO-derived RGC maybe useful to the study of the mechanisms underlying the pathology of diseases involving RGC death, such as glaucoma and drug screening of neuroprotective or neurotrophic factors in vitro. Furthermore, purified RGCs could not only lead to future personalized medicine by using patient derived iPSCs, but also might be useful for transplantation replacement therapy.^179,180^

Despite the extensive further research needed, the introduction of RO technology offers an excellent ex vivo platform which is capable of recapitulating major molecular and cellular events of human retinogenesis in vitro.^20^ The combination of RO generation with bioreactor technology will permit the scaling-up of the process,^182^ a demand that will have to be met in the near future, given the requirement of high cell numbers needed for cell-based therapies and drug-screening approaches.^20^ RO technology represents a milestone in the field of retinal research. It is the first in vitro culture system that allowed for the generation of clinically relevant numbers of all retinal cells, including photoreceptors, necessary for the development of future cell-based therapies^20^ and have never been achieved previously using traditional 2D culture techniques.^18,183^

Retinal organoid systems closely model native developmental processes, and present maturing organised structures of varying cell types.^41^ Such systems can be maintained for long periods of time whilst preserving their histological features, allowing cells to reach a developmental stage comparable to that at which functional maturation of the photoreceptors begins in the human retina.^41^ This represents a reliable model, more physiologically relevant to study the developmental and early human retinal cell fate decisions, high throughput drug screening and regeneration and cell replacement.^179^ ROs derived from patient-specific hiPSCs can also serve as useful disease modelling and progression tools. Up-scale manufacture of cells for clinical use can also be aided by freezing organoids at different stages throughout their maturation, thereby presenting higher numbers (different ratios) of cell types, for example, RCG therapies will be fulfilled with earlier stage ROs. It has been demonstrated that differentiating organoids can be cryopreserved whilst preserving their integrity, maintaining the cellular viability and differentiation capacity, being able to express retinal markers and develop mature photoreceptors following thawing.^168,184^ Moreover, although cryopreservation of isolated photoreceptors directly following magnetic-activated cell sorting (MACS) has resulted in significant decreases in cell viability, MACS could be performed on thawed organoids resulting in a small, but not significant, increase in the number of apoptotic cells.^185^ This would result in efficient enrichment of photoreceptor precursors prior to transplantation.^10^ Options of cryopreservation facilitates the quality control of the organoids with validation at intermediate stages, ensuring the possibility to store the organoids at the appropriate stage of differentiation for downstream applications, passing technical limitations in the generation of the large number of cells required for transplantation.

RO technology is still in a developmental phase and crucial research is still required for this field to advance. A fundamental requirement in the development of RO and thus cell-based therapies, is the establishment of robust protocols that allow the derivation of large numbers of donor cells from a renewable source that recapitulate the characteristics of the endogenous cell types they are designed to replace. Currently, the variances between protocols and cell lines used, coupled with the use of different analytical techniques, or studied markers, generate massively different sets of data that do not allow studies to be entirely reproducible and/or directly comparable. For example, studies normally present one portion of the retinal organoid as an example of the retinal stratification/marker expression, assuming that it is representative of the full organoid. A major goal in the community should be the generation of a standardised, reliable, and robust protocols that will allow studies to be directly compared, and more importantly reduce the differentiation and maturation heterogeneity between organoids and protocols to be Good Manufacture Practice (GMP) compliant to allow the scale-up production for future translation of therapies.

Despite significant advances, total maturation of generated tissues currently does not occur. ROs do not present fully mature photoreceptor outer segments. It has been proposed that this hampered maturation may be due to the misplacement of RPE and the lack of important physiological interactions between matured photoreceptor and RPE cells in ROs.^186^ The RPE is essential for normal retinal function, including phagocytosis and processing of shed-out photoreceptor outer segments and secretion of neurotrophic and vasculotrophic growth factors.^31,168^ A polarized and functional RPE is vital for the survival of photoreceptors in vivo and a crucial part of the visual cycle. Although RPE misplacement does not prevent the formation of the retinal organization, it may prevent the formation of the proper lamination and maturation of the cells.^40,41^ Results seem to suggest that, although contact with RPE might not be necessary for photoreceptor development,^40,41^ diffusion of its factors facilitates their differentiation, as optic vesicles maintained in RPE conditioned medium demonstrate improved lamination. In contrast, Akhtar et al.^186^ showed that whilst RPE-RO direct contact enhanced the differentiation process of the organoids, leading into an earlier differentiation of photoreceptors, RPE conditioning media did not affect their development. This supports the idea that direct contact between the RPE and ROs is necessary. Furthermore, since ROs are not exposed to 11-cis retinal, a necessary component for phototransduction, they may be less physiologically active and thus less susceptible to injury.^40^ Future studies are required to address these limitations.

Limitations in oxygen, nutrient, and waste exchange, mean that organoids have a limited growth potential. Lack of vascular circulation can induce hypoxia during organoid culture and often cellular necrosis occurs in the centre of organoids which consequently hinders the normal development and maturation of neurons.^48,187^ This heavily limits the continuous growth and long-term maintenance of functional cells in organoids. Nonetheless, vascularization is an issue in tissue engineering as a whole and novel approaches are in constant development.^19^ Recently Shi et al.^187^ co-cultured hESCs or hiPSCs with HUVECS in vitro to generate vascularised human cortical organoids (vOrganoids). These organoids demonstrated mesh-like or tube-like vascular systems which, although no real blood cells were present, may have supplied greater levels of oxygen and nutrients for the cells, promoting cell proliferation and differentiation and preventing cell death, which could account for the larger size and enhanced neurogenesis present in vOrganoids. Results suggest that neurons within the vOrganoids were more mature, exhibited increased dendritic or axonal growth, and expressed fewer levels of apoptosis/hypoxia markers than those in non-vascularized organoids. Although the retinal vascular system is completely different to the cerebral system, given the limitations of traditional organoid culture, similar approaches applied to RO culture may solve the problems of insufficient oxygen supply and nutrient support to organoids to some extent. This has potential to enhance the maturation and functional development of retinal cells, particularly photoreceptors, in RO culture.

Importantly, ROs have not yet shown irrefutable evidence of neuron functional connexions and synapses, and maturation of current cultures. Further studies are necessary to assess if the transplanted photoreceptors can develop true synaptic connections that are able to transmit visual information, and if they can survive into the host retina and for how long.^188^ To date, studies have only been able to suggest this due to the expression of a variety of synaptic and phototransduction cascade involved proteins,^40,41,157,170^ electrophysiological responses from whole-cell patch clamp voltage recordings,^40,41^ and calcium imaging^39^ showing that grafted photoreceptors presented comparable membrane depolarization-induced outward currents and intracellular calcium oscillations upon chemical stimulation.^189^ At present, these results are not robust enough to fully understand the level of functionality of the cells, and if the in-situ RO neural networks are functional and able to transmit visual information.

A variety of imaging modalities have been used to assess the real-time changing metabolic state and the development of RO.^190^ Live imaging techniques enhance the understanding of developmental and metabolic changes during RO maturation, and as opposed to histologic analysis, provide a non-invasive approach to interrogate engineered tissue systems in basic research and cell therapy settings.^190^ Similarly, pursuing to improve the quantitative analysis of organoid function for use in drug discovery, Vergara et al.^47^ developed a screening platform that uses fluorophore technology to assess live RO development and physiology. This versatile platform is based in a microplate reader that assess xyz-dimensional accurate quantification of fluorescent reporters. It was used to study RO development in a quick, accurate, and reproducible manner and has the potential of being extrapolated to other complex organoid systems. Development of similar technologies are of great importance within the field as they can overcome the inherent variability issues and a general lack of robust quantitative technologies for analysing organoids on a large scale, which generate severe limitations for their use in translational applications. Live imaging techniques can enhance the utility of RO as development and disease models, drug screening tools, or sources for retinal cell replacement therapies.

## Microfluidic technology

Despite the outstanding capacities of 3D scaffolds and organoids for the recapitulation of the architectural 3D microenvironment, controlling culture conditions in terms of drug delivery and oxygen/nutrient supply, as well as adequate data acquisition and monitoring are currently highly limited.^191^ Microfluidic technology represents one of the most exciting developments at the interface between biology and engineering.^46^ This approach has been well-used for neuronal populations of various kinds (as well as many other organ/tissue systems) although there have been limited reports of microfluidic retina-on-a-chip systems. Here we briefly review the technology and draw attention to future areas where retinal tissue engineering may benefit.

Microfluidic chips are platforms that allow the microscale precise allocation of single cells in a highly complex and organised 2D-2.5D systems, breaking down the inaccessible complexity inherent to 3D tissue engineering techniques. Rather than the recapitulation of a whole organ, microfluidic devices aim for the recapitulation of its structure and function through the combinations of two or more tissues (cell types), recreating tissue–tissue interfaces, exposing them to their physiologically relevant chemical and mechanical microenvironments.^36,46^ Cells can be compartmentalized and independently modulated, separated by microchannels, valves, permeable membranes and hydrogels.^192^ Typically, these devices are built on a flat solid substrate which is bonded to a polydimethylsiloxane (PDMS) block containing the compartments and microchannels.^36^

The basic configuration is a microfluidic chip composed of two independent chambers connected by an array of microchannels (Figure 4), which presented spatial and fluidic segregation of neuronal soma from axons^33^ while allowing interaction between the neuron populations.^193^ This simple, yet pioneering basic configuration has set the base and key reference for modern microfluidic neural studies, permitting future modifications by adding more chambers and increasing the complexity of the neural models.^194,195^ This design has been widely used in co-culture studies of different CNS neurons or glia cell, including cortical-cortical and cortical-thalamic, hippocampal-glia, and cortical neurons-genetically modified astrocytes,^34,196–200^ Others have specifically studied cell behaviour affecting neurite outgrowth and guidance,^96^ dendritic spines and synapses formation.^200^ The posterior addition of a perfusion chamber perpendicular to the microchannels in 2010 by Taylor et al.^201^ allowed the visualization and manipulation of synaptic, presynaptic, and postsynaptic bodies and the study of differential dendrite/axon extension. Different groups have aimed to promote unidirectional connectivity (transmission of information) of neurons,^34,202^ and the ability to generate uni- and bi-directional neural connections has been demonstrated.^96^

Diffusion of cell signalling molecules and cytokines biomolecules can be spatio-temporally controlled between device ‘*ports*,’ allowing the generation of diffusion-based gradients for example.^92,191^ Devices can be equipped with optical and electrical sensors which allow the acquisition of more precise measurement of cell behaviour than those that can be obtained during in vivo or in vitro static models.^11,36,44^ Furthermore, the planar presentation facilitates real-time imaging in a non-destructive, label-free and easily applicable manner.^36^ Inclusion of electrodes or probe dyes, parameters such as oxygen concentration, pH or glucose consumption, can be monitored directly to study changes in cellular metabolism.^11^ The intra and extracellular processes in response to specific stimuli can be quantified at a single cell and high temporal resolution (<1 s) over prolonged cell culture periods,^191^ allowing high throughput analysis of the biological systems.

Incorporation of an active and tuneable vascular system enables not only the analysis of cell secretions, transcriptions, and protein expression through designed compartmentalisation but also, enables diffusion of nutrients and waste products necessary for the maintenance and growth of the tissue. This solves one of the major problems observed with 3D bioprinted constructs and retinal organoid technology. Indeed, the key feature of microfluidic platforms is the manipulation of fluid flow to define the culture environment.^12^ Aspects such as perfusion flow, ECM/material organization, spatial control over cell patterning (and co-culture), and soluble factor and biochemical cue gradients can be achieved *via* microfluidic platforms.^203,204^

Microfluidic platforms have been used to address specific research questions extending from axonal guidance, synapse formation, or axonal transport to the development of 3D models of the CNS allowing for pharmacological testing and drug screening ^33^; however, the application of microfluidics within retinal tissue research has been scarce. Segregated neurite outgrowth was first reported in seminal studies using the Campenot chamber, showing isolated neural projections and connections between cell populations.^205–207^ Whilst the models were able to demonstrate the elongation of neurites with separated/isolated cell bodies, they were limited in that only a single cell type was used across all the populations. More recently, a variety of microfluidic cell co-culture platforms have been developed in order to study controlled cell-cell interactions and organizing different cell lines to recapitulate the function of tissues or even organs. For example, reconstruction of the complex basal ganglia mid-brain circuitry central to Parkinson’s disease was modelled with a five-cell type device enabling in situ assessment of functional activity in each population and importantly across the whole cell network.^34,35^

The combination of *lab-on-a-chip* technology with specific organ characteristics, such as interstitial fluid flow (osmotic pumps) in the case of the brain, makes it possible to create organ (brain)*-on-a-chip* platforms.^208^ *Organ-on-a-chip* devices can replicate fundamental aspects of animal physiology essential for the understanding of drug effects, improving preclinical safety and efficacy testing.^11^ Park et al.^209^ demonstrated that the neural network formation in neurospheroids was significantly reinforced by fluidic flow on a microfluidic device. Their study shows how integration of spheroids and microfluidic technology yielded a 3D in vitro model with more relevant physiological outcomes. Johnson et al.^210^ 3D printed a nervous system on a chip for the study of viral infection in the nervous system. It mimicked the critical function of glial cell–axon interfaces in the nervous system containing multiple neural cell types. Thus, *body-on-chip* devices are integrated systems with multiple cellular micro-environments/organoids that can be designed to simulate the systematic function of the body and to predict the pharmacokinetics of drugs in preclinical studies to enhance the results of human clinical trials.^11,44^ Nonetheless, *on-a-chip* device studies can generate high throughput measurements at lower cost and with fewer biological resources than traditional systems, which require large amounts of culture media and millions of cells versus a few millilitres and several thousand cells per tissue in microfluidics.^11^

Although there are many reports of microfluidics or ‘*on-a-chip*’ technologies being used for neuronal investigation, this has not broadened to include retinal work as much as peripheral nerve or brain circuit formation. However, a pioneering study that combines the biological self-assembly capabilities of ROs with the precisely controllable assembly and measurements of microfluidic platforms was reported.^39^ This work presented a novel device as a micro-physiological model of the human retina in which the lack of vascularisation and co-location with PRE limitation were successfully addressed. The apposition of a hyaluronic acid-based hydrogel embedded mature RO with a monolayer of RPE cells has enabled for the first time the recapitulation of the interaction between mature photoreceptor outer segments and RPE in vitro (Figure 1(c)). Previous attempts have failed to recapitulate the precise RPE-photoreceptors arrangement, resulting in unpredictable and unorganized RPE formation during RO generation, and thus, fail to yield fully mature outer segments. This was successfully achieved and more importantly, demonstrated that RO-RPE interactions enhance the formation of outer segment-like structures and the establishment of in vivo-like physiological processes including shed-out outer segments phagocytosis and calcium dynamics never seen before in RO.^39^ The number of outer segment structures was approximately three times higher in the RPE-RO chips compared to the chips without RPE. Although this retinal-microfluidic device showed promise as a disease/drug testing model, there were limitations in the long-term survival and maturation of cells due to lack of vasculature.

There has been significant research into the applications and development of microfluidic platforms. More in-depth reviews of *organ-on-a-chip* platforms latest developments, covering the use of materials, cells, the chips technologies and limitations for the different body tissues and future perspectives can be found elsewhere.^11,44^ Neto et al.^33^ review the development of microfluidic platforms with a focus on the CNS and peripheral nervous system cells and their interactions with other cells types. Other reviews also cover the necessary considerations for in vitro modelling design, along with recent advances from 2D culture systems to 3D organoids and bio-artificial organs.^208,211^

## Is retina-on chip technology on the horizon?

Although important knowledge has been gained regarding CNS neurons, their behaviour and synaptic formation relating to normal and diseased tissue, research using retinal neurons remains in its infancy by comparison. Microfluidic platforms provide a useful tool for the study of the synaptic connection and cell behaviours in the retina not available through other methodologies. Furthermore, microfluidic chips can be used to gain knowledge into the cellular communication and functional integration between donor and host retinal cells in the context of cell transplant therapies.^192,212^

Microfluidic chips have been investigated in the field of the ocular sciences, however, none of them have yet fully focussed on neural retinal cell networks and its functionality. Different groups have used microfluidic chips as a tool to: investigate the localized and controlled application of drugs or signalling molecules^35^ explore neurotransmitter stimulation of the retina, as a better alternative to electrical retinal prostheses,^213–215^ model the pathophysiology of glaucoma and its effects on RGC axon and total neurite length, cell body area, dendritic branching, and cell survival; and as a platform for the screening of potential pharmacological agents for neuroprotection,^216^ or to generate *cornea-on-a-chip* platforms to model evaporative dry-eye disease for high-content drug screening^22^ and to study topically applied ocular drug absorption and permeation.^217,218^ Others have used microfluidics platforms to recapitulate the pathogenesis of choroidal neovascularization as a model of *Wet-AMD-on-a-chip.*^37^ This has facilitated the engineering and configuration of 3D vascular networks that could be used to model ocular angiogenesis,^219^ and to study how alterations in glucose concentration and/or oxygen level affects the secretion of VEGF by RPE cells (ARPE-19) in an angiogenesis microfluidic device by co-culturing the RPE and HUVECS.^220^ Such studies highlight how microfluidic platforms can be physiologically representative in vitro ocular models, and relate to choroid neovascularisation.^221^ Yeste et al.^36^ developed a novel microfluidic device whereby cells are arranged in parallel compartments but are highly interconnected through a grid of microgrooves located under the cells. They co-cultured primary human retinal endothelial cells, a human neuroblastoma cell line and a human RPE cell line to model the tissue-tissue interface of the retinal blood-barrier. Adbolvand et al.^222^ reported the first study showing continuously perfused long-term retinal differentiation of hiPSCs within a microfluidic device, demonstrating that convective delivery of nutrients *via* perfusion plays a significant impact upon the expression of key retinal markers.

Marivel Vazquez’s group have developed different microfluidics platforms to study retinal precursor cell migration dependant behaviour upon chemical concentration gradients of stromal derived factor (SDF-1) within the geometry of the human and mouse retina,^223^ and combined electro-chemotactic fields,^212^ and Müller glia cell migration response to a variety quantitatively-controlled microenvironments of signalling factors implicated in retinal regeneration (basic FGF, FGF8, VEGF; and Epidermal Growth Factor, EGF).^224^ When incorporated into current transplantation therapies approaches, Vazquez’s findings would not only improve the transplantation outcomes, severely limited by the low numbers of donor cells able to migrate and integrate into the damaged retinal tissue, but also, serve to develop novel migration-targeted treatments.

Regeneration of the retinal structure is a promising and foreseeable, yet unestablished treatment strategy for retinal diseases.^221^ The differentiation and maturation of all the retinal cell types together with the development of their synaptic connections, is necessary for functional retinal regeneration. However, although microfluidic technologies have been extensively used to explore different aspects tackling retinal regeneration in vitro, none have presently aimed for the reconstruction of a full functional retina. Su et al.^225^ were the first and only group to date to use a microfluidic system for the culture and study of retinal neural cells. Their system composed of the basic configuration of two independent chambers connected by an array of 50 microchannels of variable sizes. They showed how microchannels with width <5 μm allowed axon and dendrite growth whilst blocking the main cell body isolated. Three days after cell seeding, retinal cells extended their axons and dendrites throughout the connecting microchannels and made structural and functional synaptic connexions between the separated neurons. Cell synapses were localised and quantified by not only microchannel occupation, but also by immunostaining of RGC marker (β III-tubulin) and synaptic marker CD95. Furthermore, functionality of the connection, and so, electrical communication between retinal precursors, was evaluated by fluorescence imaging of phosphorylated extracellular signal-regulated kinase of retinal precursors.

In summary, Su et al.^225^ demonstrated that their ‘*retinal synapse regeneration chip*’ permitted retinal neurons to form oriented and functional synaptic connections. This system might be therefore further developed and investigated not only as a more physiologically relevant model for the study of inhibitory and excitatory molecules on the dynamics of synaptic regeneration and cells crosstalk but also, as a control model for RO-derived cell replacement therapies. Despite their promising reported results, some factors should be considered in the future. Su et al., used R28 retinal progenitor cells, not directed to any specific cell fate (only expressing a RGC marker). R28 cells have only been shown to differentiate into RGC, Müller glial cells and photoreceptors.^52,226,227^ Future studies will hopefully address the fabrication of a more complex and physiologically relevant *retina-on-a-chip.* Inclusion of the seven principal retinal cells, or at least the main functional unit of photoreceptors, bipolar and RGC should be a step in the right direction. Ideally, new microfluidic devices will allow for the co-culturing of retinal cells in separated but ordered chambers mimicking the natural retinal layers spatial allocation, allowing formation of functional synapses and transmission of the information as it would happen in the in vivo environment.

## Conclusion

The development of new technologies together with the ever-increasing knowledge regarding cell biology and behaviour have made possible the advancement of biomedical sciences up to a state unimaginable 50 years ago. While bioprinting allows for the construction of anatomically and physiologically accurate 3D biological structures, it is also prepared to advance the drug development process. 3D human tissue models, may precede animal trials to test for efficacy and toxicity. Despite the advantages, several limitations inherent to each technological approach restricts current research and as such, retinal degenerative diseases are yet to be solved. A combination of the 3D cell position arrangements that microfluidic techniques offer, together with the number of cells and cell types that RO provides, hold great promise of revolutionizing the field of ocular sciences in the following years and will pave the way for new and revolutionary therapies in the clinic. Nonetheless, microfluidic platforms offer a versatile and non-destructive platform to analyse a wide variety of cellular responses and processes withing the construct. Importantly, they can be used to unequivocally verify the functionality of the RO-derived cells constructs or the effect of therapies such as optogenetics at a cellular level; requirements that will have to be met before advance of such therapies to the clinic. Current methodologies do not provide enough data to unequivocally evaluate the engineered photosensitive capacity of the cells after transduction and due to the lack of current clinical trials in humans, it cannot be known for certain how the regained visual capacities will be interpreted by the patients’ brain. There is still a long way to go until it a functional 3D retina can be generated in the lab, but promising results and technological advances in tissue engineering are slowly paving the way. With advances in technology and understanding of the biological niche environments within the retina, there will be major improvements in our ability to produce organised, functional neural cell circuitry *on-a-chip*, and this will drive support for cellular therapies.
